# Methotrexate-Associated Lymphoproliferative Disorder in a Patient with Psoriasis: A Case Report and Review of the Literature

**DOI:** 10.1155/2022/7178065

**Published:** 2022-04-30

**Authors:** Carolina Afonso, Adriana Roque, Cátia Almeida, Maria Beatriz Pimentão, Maria José Julião, Rodolfo Silva, Catarina Geraldes, Marília Gomes

**Affiliations:** ^1^Hematology Unit, Hospitais da Universidade de Coimbra, Centro Hospitalar e Universitário de Coimbra, Coimbra, Portugal; ^2^Pathology Unit, Hospitais da Universidade de Coimbra, Centro Hospitalar e Universitário de Coimbra, Coimbra, Portugal; ^3^Nuclear Medicine Unit, Hospitais da Universidade de Coimbra, Centro Hospitalar e Universitário de Coimbra, Coimbra, Portugal

## Abstract

Iatrogenic immunodeficiency-associated lymphoproliferative disorders (LPDs) are heterogeneous clinicopathological entities developing in patients receiving immunosuppression. Outside the posttransplant setting, methotrexate (MTX), a drug commonly used for the treatment of autoimmune diseases, is an immunosuppressive agent frequently reported to be associated with LPD. MTX-associated LPD (MTX-LPD) includes a spectrum of lymphocytic proliferations, ranging from polyclonal hyperplasia to malignant lymphoma. MTX-LPD diagnosis can be challenging, as signs and symptoms are often nonspecific and may overlap with those of several other conditions, including exacerbation of the underlying autoimmune disease. Spontaneous regression of LPD after MTX discontinuation is characteristic of MTX-LPD, therefore avoiding chemotherapeutic intervention in a significant proportion of patients. Other cases, however, should receive chemotherapy.

## 1. Introduction

Autoimmune and inflammatory rheumatic diseases have been associated with an increased risk of LPD, related to the chronic antigenic stimulation and immune dysregulation underlying those diseases [1]. Additionally, immunosuppressive agents frequently used to treat these diseases, such as MTX, have also been suggested to play a role in lymphomagenesis [2–4]. As a result, the World Health Organization (WHO) recognizes lymphoproliferative disorders arising in patients treated with immunosuppressive drugs for autoimmune disease as a separate nosological entity classified as “other iatrogenic immunodeficiency-associated LPDs” (OIIA-LPD) in the revised 4th edition of the WHO Classification of Tumors of Hematopoietic and Lymphoid Tissues [4].

Most OIIA-LPD develop in patients treated with MTX for autoimmune diseases [5]. Therefore, MTX may further increase the risk of LPD in this population. Regarding rheumatoid arthritis (RA), for example, it is estimated that patients undergoing treatment with MTX are 1.7 times more likely to develop LPD than those not treated with this drug [6, 7].

Most MTX-LPD cases are diffuse large B cell lymphoma (DLBCL; 35–60%), followed by classical Hodgkin lymphoma (CHL; 12–25%), while other lymphoma subtypes are rare [4, 8]. Their histopathological features are similar to those found in immunocompetent patients with equivalent lymphoma subtypes [8]. Polymorphic or lymphoplasmacytic infiltrates have been described in as many as 20% of cases of MTX-LPD [4].

The pathogenesis of these entities is not entirely understood; studies suggest that the suppression of immune surveillance by MTX, due to excessive inhibition of T helper 1 CD4+ T cells and effector memory CD8+ T cells, plays an important role [3]. In addition, by inhibiting CD8+ Epstein-Barr virus (EBV)-specific T cells, MTX indirectly promotes proliferation of latent EBV-infected B cells, which are normally suppressed [3, 9, 10]. Indeed, these MTX-LPD are often EBV related: EBV is almost always found in polymorphic infiltrates, in approximately 80% of MTX-CHL and in 25–60% of MTX-DLBCL. [4].

Spontaneous regression of LPD after MTX discontinuation is characteristic of MTX-LPD, occurring in approximately 50% of cases [3, 11]. Patients with nonregressive lymphomas and those with relapse/regrowth (R/R) events require histology-adjusted chemotherapy [5].

## 2. Case Report

A 56-year-old male patient diagnosed with moderate-to-severe plaque psoriasis at the age of 14 had been receiving treatment with low-dose MTX (15 mg per week) for more than one decade when he developed constitutional symptoms of fatigue and unintentional weight loss (approximately 20% of his normal bodyweight). Physical examination revealed multiple palpable bilateral supraclavicular and cervical lymph nodes, as well as a palpable spleen 3 cm below the costal margin. Laboratory investigation revealed white blood cell and platelet count, mild anemia (hemoglobin 11.1 g/dL), elevated lactate dehydrogenase (314 U/L, normal range (NR) < 250), and increased cholestatic enzymes (*γ*-glutamyltransferase 192 U/L, NR < 55 U/L; alkaline phosphatase 237 U/L, NR 30–120 U/L). Microbiological and serological tests did not reveal active infection.

**Figure 1:**
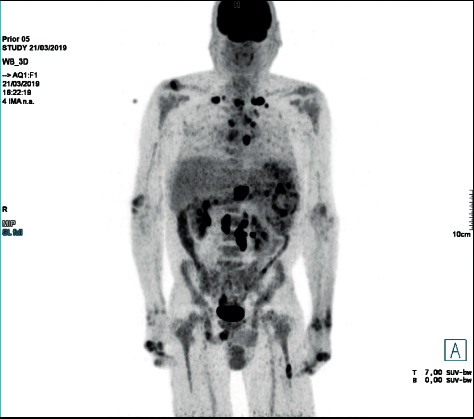
18F-FDG PET/CT performed at initial presentation showing hypermetabolic involvement of the spleen and lymph nodes above and below the diaphragm, as well as small, 18F-FDG-avid pulmonary nodules and focal skeleton/bone marrow hypermetabolic involvement.

Computed tomography (CT) scan of the chest, abdomen, and pelvis showed mediastinal and paraaortic lymphadenopathy (the short-axis of the largest lymph node was 29 mm) and splenomegaly (with a 210 mm longitudinal axis). Additionally, the patient performed a ^18^F fluorodeoxyglucose positron emission tomography/CT (^18^F-FDG PET/CT) which revealed extensive hypermetabolic involvement of the spleen (with a maximum standardized uptake value, SUV max, of 16.7) and lymph nodes above (cervical, supraclavicular and mediastinal lymphadenopathy) and below the diaphragm (paraaortic, celiac, hepatic hilar, and peripancreatic); additionally, there was evidence of small, ^18^F-FDG-avid pulmonary nodules and focal skeleton/bone marrow hypermetabolic involvement (Figure 1).

As most lymph nodes were either small or not easily accessible for biopsy, a CT-guided percutaneous core biopsy of focal splenic lesion was performed; however, histologic examination found normal splenic tissue. Bone marrow biopsy aspiration and trephine biopsy also did not detect clonal lymphoid infiltration. Given the high clinical suspicion of MTX-LPD (and despite the absence of histological confirmation), MTX was discontinued, and a watch-and-wait strategy was adopted. Constitutional symptoms progressively improved, reinforcing the diagnostic hypothesis. Since then, psoriasis was managed with combinations of topical agents and psoralen plus ultraviolet A (PUVA) therapy.

**Figure 2:**
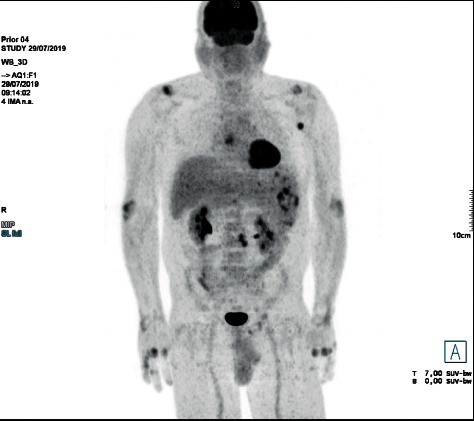
18F-FDG PET/CT performed four months after MTX discontinuation, showing a reduction of the hypermetabolic activity of spleen and lymph nodes, and a spontaneous resolution of the hypermetabolic involvement of lungs and bone marrow.

Four months after MTX discontinuation, a new PET/CT scan was performed, showing a decrease in size and metabolic activity of supra and infradiaphragmatic lymphadenopathy, a reduction of the hypermetabolic activity in the spleen (SUX max of 9.5), and a spontaneous resolution of the hypermetabolic involvement of the lungs and bone marrow (Figure 2). Given the partial regression of the disease and the absence of constitutional symptoms since MTX was withdrawn, close surveillance was maintained.

A PET/CT scan performed five months later (nine months after MTX cessation) revealed an increase of the hypermetabolic activity in several supra and infradiaphragmatic lymphadenopathies accompanied by a paradoxical reduction in the size of the lymph nodes; there was also evidence of multiple splenic lesions with an increased hypermetabolic activity (SUV max of 13.2). Given the imagiologic evidence of progressive disease, a CT-guided percutaneous core biopsy of the spleen was repeated, but a definite diagnosis could not be established due to unrepresentative tissue sampling. Since the patient was asymptomatic at the time, a watchful waiting approach was maintained.

**Figure 3:**
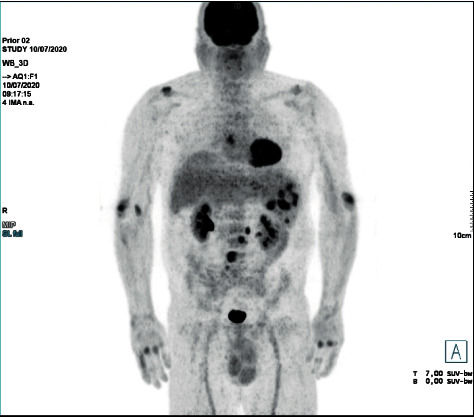
18F-FDG PET/CT performed 15 months after MTX withdrawal with an increased hypermetabolic activity in the spleen and a new hypermetabolic lesion involving the L4 vertebral body, consistent with progressive disease.

Six months later (15 months after MTX cessation), another PET/CT scan was consistent with progressive disease, with development of a new hypermetabolic lesion involving the L4 vertebral body, as well as an increased hypermetabolic activity in the spleen (SUV max of 15.9) (Figure 3).

**Figure 4:**
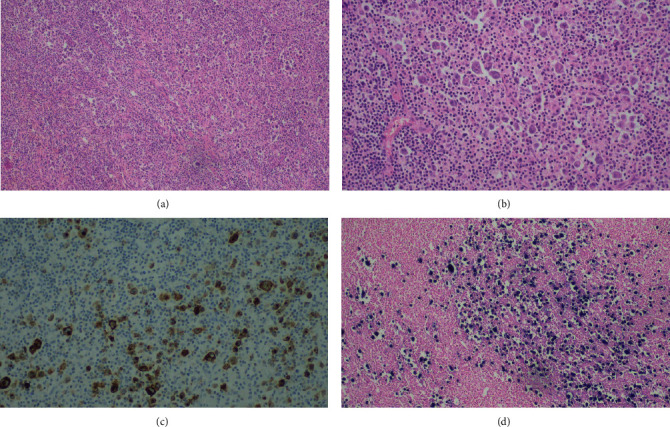
Histopathological examination of splenectomy specimen. Infiltration of the splenic tissue by large pleomorphic neoplastic cells resembling HRS cells: (a) H&E stain, 100x magnification; b) H&E stain, 200x magnification]. These cells were positive for CD30 (c) and EBER (d).

The patient was then proposed for diagnostic splenectomy. At macroscopic examination, the splenic parenchyma showed multiple nodules, the largest one with approximately 4 cm diameter, located near the splenic hilum. Histopathological analysis revealed multiple parenchymatous nodules composed by numerous Hodgkin and Reed-Sternberg (HRS) cells positive for CD30 and CD15, with dim expression of PAX5, and negative for CD45, CD20, CD10, and ALK; these cells were also positive for EBV-encoded RNA (EBER) (Figure 4). Immunoglobulin heavy chain (IGH) clonality testing was not performed.

A diagnosis of MTX-associated CHL (MTX-CHL), Ann Arbor stage IV-BS, advanced stage by the German Hodgkin Study Group (GHSG), International Prognostic Score (IPS) 3, was established. The patient started treatment according ABVD protocol (adriamycin, bleomycin, vinblastine, and dacarbazine). An interim PET/CT scan (after two cycles) showed a complete metabolic response (Deauville score 3), and the patient proceeded to four additional cycles of chemotherapy with omission of bleomycin (AVD), as per protocol. End-of-treatment PET/CT was consistent with a complete metabolic response (Deauville score 1). At the time of writing (eight months after completing the treatment), the patient does not show evidence of relapse and/or late treatment-related toxicity.

## 3. Discussion

Patients with autoimmune diseases, such as rheumatoid arthritis and psoriasis, are at increased risk of developing LPD [12]. For RA, for example, the risk of lymphoma is 2–4 times higher than that observed in the general population, and this risk that may be slightly increased by treatment with MTX [6, 7].

Diagnosis of MTX-LPD can be challenging, given that patients may present with nonspecific constitutional symptoms, which can arise from a variety of conditions, including exacerbation of the underlying autoimmune disease, infections, and malignancies. Additionally, given the heterogeneous pathology of MTX-LPD, multiple or repeated biopsy may be needed to establish a diagnosis [13]. Importantly, no association has been found between the duration and/or dose of MTX and MTX-LPD development [14].

In the case described above, although there was already a high index of suspicion of MTX-LPD (given the improvement of clinical symptoms and regression of disease after MTX withdrawal), histological confirmation took 21 months, mainly due to repeated nondiagnostic biopsies.

In our case, EBV was detected by in situ hybridization, in accordance with previous studies which report a high incidence (approximately 80%) of EBV positivity in CHL-LPD [4, 15].

Approximately 40–50% of MTX-LPD cases occur at extranodal sites such as the skin, digestive tract, liver, spleen, lung, bone marrow (BM), and central nervous system (CNS) [4, 11]. Regarding MTX-CHL, several studies reported higher rates of advanced-stage disease and extranodal disease when compared to sporadic CHL [8, 16–18]. Indeed, our patient presented advanced-stage disease with extranodal involvement, including the spleen, lungs, and BM.

In patients with sustained regression of MTX-LPD after immunosuppression withdrawal, chemotherapy may be avoided [11, 19]. Therefore, the first step if MTX-LPD is suspected is MTX discontinuation [18]. For most patients achieving complete remission (CR), this is expected to occur within a time interval of 4 weeks after discontinuation of immunosuppressant; conversely, for patients experiencing a partial remission (PR), the time interval between discontinuation of immunosuppressive treatment and clinical response is usually longer, often occurring about 2-3 months later [20]. In a retrospective study, the EBV-positive rate in MTX-LPD patients exhibiting spontaneous regression of LPD was higher than that observed in MTX-LPD patients without tumor regression (54.5% vs. 21.1%, respectively), suggesting that EBV may be involved not only in the development of MTX-LPD but also in its regression after MTX withdrawal [21]. Histology may also influence regression rates after MTX cessation, which are reported to be lower in MTX-CHL than in MTX-DLBCL [8, 10, 22].

Even in cases of spontaneous remission, careful observation is important, as R/R of MTX-LPD has been reported in 18–45% of patients, with a reported median duration until R/R of 10.6 months (0.7–35.6) [11, 18]. In the case described above, a partial remission of LPD was observed 4 months after MTX withdrawal, but regrowth was documented after an additional 12 months off treatment.

In cases with nonregressive LPD after MTX withdrawal and in those with R/R events, chemotherapy is the treatment of choice [5]. Regarding MTX-CHL, although there is no established standard chemotherapy regimen, most reported cases were treated with ABVD, which is considered the standard treatment for sporadic CHL [13, 23]. MTX-CHL seems to have identical response rates to chemotherapy, progression-free survival (PFS), or overall survival (OS) to sporadic CHL [16].

In recent years, an increasing number of case reports regarding the use of brentuximab vedotin (BV) in combination with AVD (BV+AVD) in front-line treatment of MTX-CHL has been published, with favorable responses and an acceptable toxicity profile [22, 23]. Although it is unknown whether MTX-CHL patients are particularly susceptible to bleomycin-induced lung toxicity, this bleomycin-free regimen represents an alternative for these patients who may have underlying pulmonary complications related to their autoimmune disease [22, 23]. Although immune checkpoint inhibitors may also be suitable for patients with MTX-CHL, their safety in patients with preexisting autoimmune disease remains controversial, particularly due to the risk autoimmune disease flare [24].

## 4. Conclusion

We herein report a rare case of LPD in a patient with a past long-term oral MTX administration, who successfully attained complete remission following treatment with ABVD/AVD. The case illustrates the difficulty in establishing a diagnosis of MTX-LPD. When suspected, MTX should be discontinued, as this may lead to an improvement of clinical symptoms and a spontaneous regression of disease. The case also highlights the importance of careful observation, even in cases of spontaneous remission after MTX cessation, due to possible occurrence of R/R events, which require chemotherapeutic intervention.

## Data Availability

Data sharing is not applicable to this article and data used to support this study are included within the article.
